# Cytotoxic Activity of *Christia vespertilionis* Root and Leaf Extracts and Fractions against Breast Cancer Cell Lines

**DOI:** 10.3390/molecules25112610

**Published:** 2020-06-04

**Authors:** Joanna Jinling Lee, Latifah Saiful Yazan, Nur Kartinee Kassim, Che Azurahanim Che Abdullah, Nurulaidah Esa, Pei Cee Lim, Dai Chuan Tan

**Affiliations:** 1 Laboratory of Molecular Biomedicine, Institute of Bioscience, Universiti Putra Malaysia, Serdang 43300, Malaysia; joyousjoanna@gmail.com (J.J.L.); nurulaidahesa@yahoo.com.my (N.E.); 2 Department of Biomedical Science, Faculty of Medicine and Health Sciences, Universiti Putra Malaysia, Serdang 43300, Malaysia; 3 Department of Chemistry, Faculty of Science, Universiti Putra Malaysia, Serdang 43300, Malaysia; kartinee@upm.edu.my (N.K.K.); limpeicee@yahoo.com (P.C.L.); tandaichuan@yahoo.com (D.C.T.); 4 Department of Physics, Faculty of Science, Universiti Putra Malaysia, Serdang 43300, Malaysia; azurahanim@upm.edu.my

**Keywords:** *Christia vespertilionis*, daun rerama, root, leaf, anticancer, cytotoxicity, MDA-MB-231, MCF-7, antioxidant, alternative medicine

## Abstract

*Christia vespertilionis*, commonly known as ‘Daun Rerama’, has recently garnered attention from numerous sources in Malaysia as an alternative treatment. Its herbal decoction was believed to show anti-inflammatory and anti-cancer effects. The present study investigated the cytotoxicity of the extract of root and leaf of *C. vespertilionis*. The plant parts were successively extracted using the solvent maceration method. The most active extract was further fractionated to afford F1–F8. The cytotoxic effects were determined using MTT assay against human breast carcinoma cell lines (MCF-7 and MDA-MB-231). The total phenolic content (TPC) of the extracts were determined. The antioxidant properties of the extract were also studied using DPPH and β-carotene bleaching assays. The ethyl acetate root extract demonstrated selective cytotoxicity especially against MDA-MB-231 with the highest TPC and antioxidant properties compared to others (*p* < 0.05). The TPC and antioxidant results suggest the contribution of phenolic compounds toward its antioxidant strength leading to significant cytotoxicity. F3 showed potent cytotoxic effects while F4 showed better antioxidative strength compared to others (*p* < 0.05). Qualitative phytochemical screening of the most active fraction, F3, suggested the presence of flavonoids, coumarins and quinones to be responsible toward the cytotoxicity. The study showed the root extracts of *C. vespertilionis* to possess notable anti-breast cancer effects.

## 1. Introduction

Breast cancer is one of the vastly occurring cancers worldwide, especially among women concerning after-treatment effects. In 2012, the World Health Organization (WHO) stated that breast cancer was the leading cause of death in women with 1.7 million new cases and accounting for 521,900 deaths [1]. The number of newly diagnosed patients continues to climb with an estimation of 2.1 million cases and 626,679 deaths in 2018 [2]. Despite the continual advancement and interventions of current therapeutic strategies, breast cancer is slowly moving into the category of chronic disease. The therapeutic side effect frequently outweighs the potential benefits as the vast majority succumb to the disease due to metastatic breast cancer. Furthermore, the fuel for each breast cancer type varies, making each case unique and posing multiple challenges in treatment. This incurs a need to discover a safer alternative to cancer control and prevention.

The practice of herbalism through medicinal plants is not foreign. Potential therapeutic plants are believed to possess good antioxidant strength which subsequently contributes toward the anti-cancer and anti-inflammatory abilities especially toward breast cancers [3,4,5]. Moreover, plant-based bioactive phytochemicals are capable of inhibiting tumor cytogenesis through various means by inhibition or modification of epigenetic processes which suppresses gene initiation, suppression and progression [6]. In this regard, compounds such as polyphenols, terpenoids and alkaloids reported in plant extracts are mostly seen to demonstrate anti-proliferative and anti-cancer properties [7,8].

Antioxidants are elements that have the ability to scavenge free radicals and inhibit lipid peroxidation which shields the body system from oxidative damage [9]. In addition, antioxidant substances have also been found to protect various protein groups, cells structures and organ systems against free radical damage [10]. The overproduction of free radicals and reactive oxygen species (ROS), however, generates oxidative stress at cellular levels, causing potential cell damage which leads to various diseases and disorders including cancers [11]. Natural-based antioxidants from a plant origin are seen as a promising approach as they are less toxic and more effective [12]. The secondary metabolites especially phenolic compounds from plants have been reported to possess remarkable reducing powers and strong radical scavenging abilities. Furthermore, natural antioxidants are able to increase oxidative capacities in the plasma preventing unwanted oxidative damage and subsequently reducing the risk of unwanted cell mutations [13].

*Christia vespertilionis* (L.f.) Bakh. F., (Family: Fabaceae) is a non-climbing perennial herb cultivated in the tropical South East Asia for ornamental appreciation. Due to its uniquely shaped trifoliate leaf, it is also locally called “Daun Rerama” or “Butterfly wing”. The plant has been traditionally used to treat tuberculosis, snake bites and promote healing in respiratory related ailments [14]. The decoction of the whole plant was believed to improve blood circulation and muscle weakness, while the topical application of the crushed leaves is capable of relieving skin conditions and healing bone fractures [15,16]. Based on recent reports, the leaves of *C. vespertilionis* were shown to exhibit anti-malarial, anti-inflammatory, anti-proliferative and anti-cancer effects against various cancer types [8,15,17,18]. Aside from that, the plant was also reported to contain a variety of phytocompounds including polyphenols, alkaloids, and terpenoids which may be constituents that are responsible for the antioxidant and anti-cancer activities [8,15,19].

In Malaysia, this plant has even been commercialized into teabags and crackers owing to its earned reputation for anti-cancer effects by herbal practitioners [20]. However, there is yet any report regarding the cytotoxic properties of the different parts of the plant extract of *C. vespertilionis* specifically against breast cancer cell lines and their antioxidant properties. The present study, therefore, investigated the possible phytocompounds responsible toward the anti-cancer activities of *C. vespertilionis* through its antioxidant properties.

## 2. Results

### 2.1. Cytotoxicity of C. vespertilionis Crude Extracts

**Figure 1:**
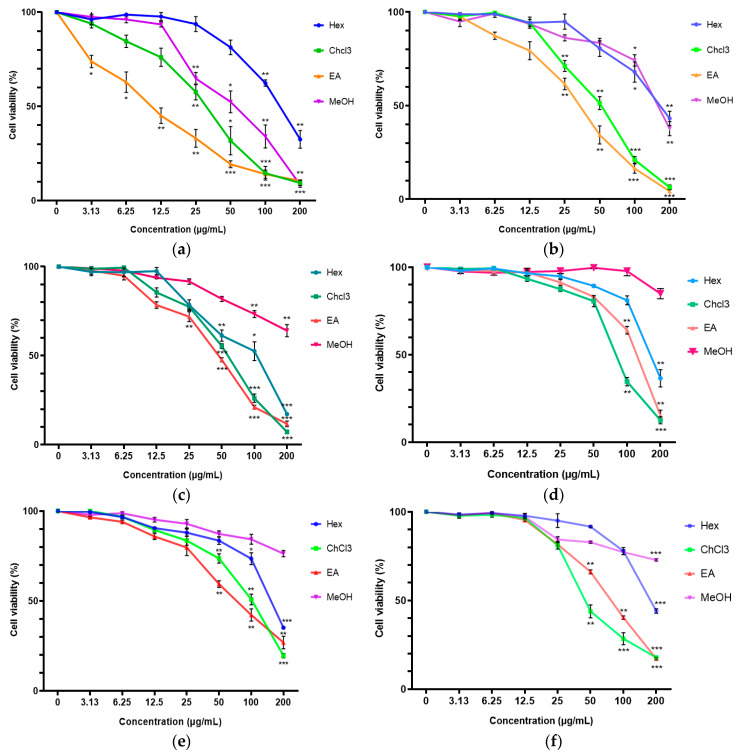
Cytotoxicity of *C. vespertilionis* extracts determined using an MTT assay; (**a**) root extracts on MDA-MB-231, (**b**) leaf extracts on MDA-MB-231, (**c**) root extracts on MCF-7, (**d**) leaf extracts on MCF-7, (**e**) root extracts on 3T3, and (**f**) leaf extracts on 3T3 cell lines. Hex, ChCl3, EA and MeOH represents the hexane, chloroform, ethyl acetate and methanol extract, respectively. All values are expressed as mean ± SD and data with asterisks are significantly different at *p* < 0.05 when compared with the untreated group (control).

Generally, the cytotoxic effects of the *C. vespertilionis* extracts were dose-dependent. As seen in Figure 1, a significant cytotoxic effect was noted from the chloroform and ethyl acetate extracts of *C. vespertilionis* against all cell lines. The inhibitory effect of the root extracts was seen to be stronger than the leaf extracts against the breast cancer cell lines, particularly toward the MDA-MB-231 cell line.

The most significant cytotoxic effect against MDA-MB-231 cell line was shown by the ethyl acetate root extract with an IC_50_ value of 11.34 ± 1.20 μg/mL followed by the chloroform root extract at IC_50_ value of 29.58 ± 3.80 μg/mL. The leaf extracts showed a similar cell viability trend against MDA-MB-231 but at higher IC_50_ values. The highest cytotoxic effects against MCF-7 cell line was also demonstrated by the ethyl acetate root extract (IC_50_ = 44.65 ± 5.78 μg/mL) followed by the chloroform root extract (IC_50_ = 54.55 ± 9.51 μg/mL), while all the leaf extracts did not show significant cytotoxicity. In the 3T3 cell line, significant cytotoxicity was recorded in the chloroform leaf extract (IC_50_ = 58.31 ± 11.07 μg/mL) followed by the ethyl acetate root extract (IC_50_ = 77.38 ± 4.71 μg/mL).

### 2.2. Cytotoxicity of Ethyl Acetate Root Fraction

**Figure 2:**
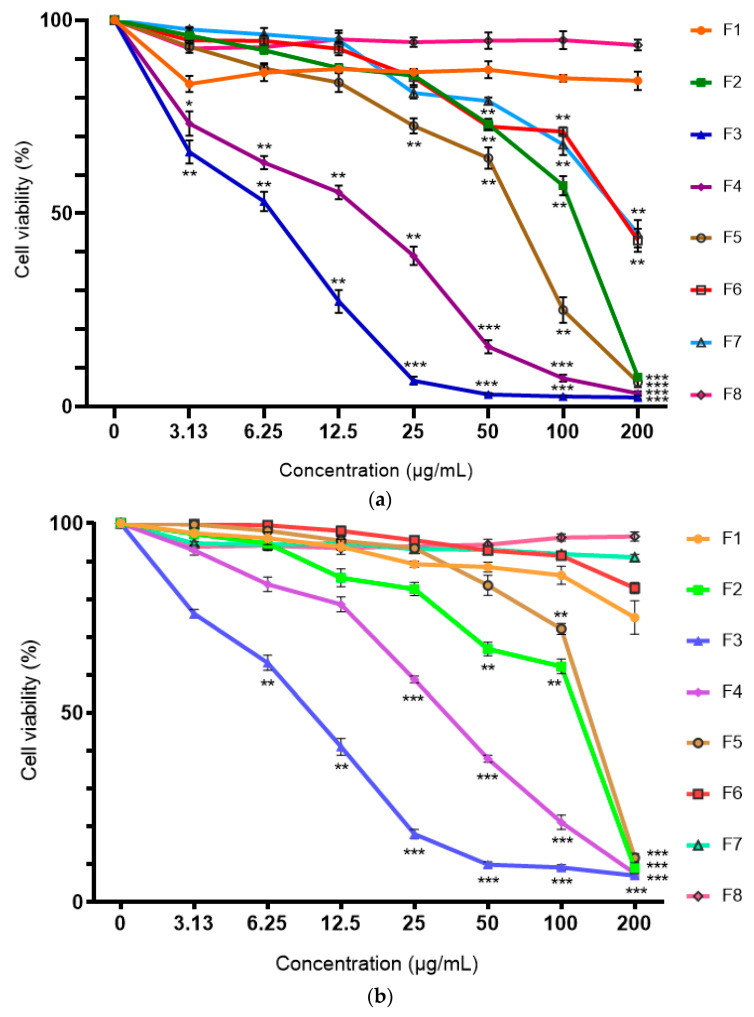

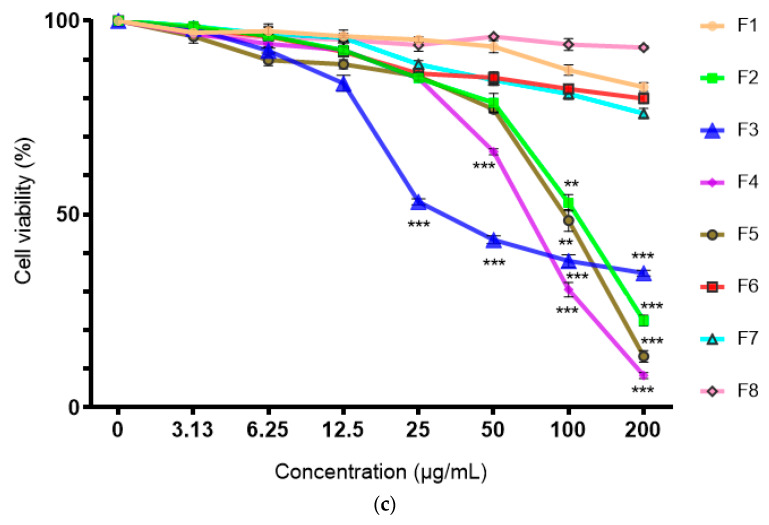
Cytotoxicity of *C. vespertilionis* ethyl acetate root fractions (F1–F8) determined using MTT assay on (**a**) MDA-MB-231, (**b**) MCF-7 and (**c**) 3T3. All values are expressed as mean ± SD and data with asterisks are significantly different at *p* < 0.05 when compared with the untreated group (control).

The ethyl acetate root extract was fractionated in increasing polarity using hexane, ethyl acetate and methanol to afford F1–F8. The cytotoxic effects of the ethyl acetate root fractions were also dose-dependent. Figure 2 shows that F3 exhibited the most significant cytotoxic effect against all the cell lines followed by F4. The inhibitory effect of F3 was found to be selective toward breast cancer cell lines but not toward the normal cell line. At the lowest concentration of 3.13 μg/mL, the cell viability of MDA-MB-231 reduced to 65.92% while the cell viability of MCF-7 cell line reduced to 76.16%. The cell viability of 3T3, however, remained at 98.01%. In addition, F3 showed the lowest IC_50_ values for both the MDA-MB-231 and MCF-7 cell lines (IC_50_ = 5.72 ± 0.99 and 8.98 ± 1.06 μg/mL, respectively) but with no significant difference. The IC_50_ value against the 3T3 cell line was recorded at 49.90 ± 8.63 μg/mL. The cytotoxicity results of the fraction suggest the potential of the F3 as an anti-breast cancer agent.

### 2.3. Selectivity Index of C. vespertilionis

The selectivity index (SI) was determined as a ratio of the normal cell line against the breast cancer cell lines. According to Kaplánek et al. [21], an SI value of more than three was considered highly selective against cancer cells. The SI analysis included the most active and second active sample from each the root extracts, leaf extracts and fractions. From Table 1, it was seen that all the active extracts and fractions exhibited selective cytotoxicity specifically against MDA-MB-231. The SI > 3 against the MCF-7 was only seen from F3.

### 2.4. Total Phenolic Content (TPC)

**Figure 3:**
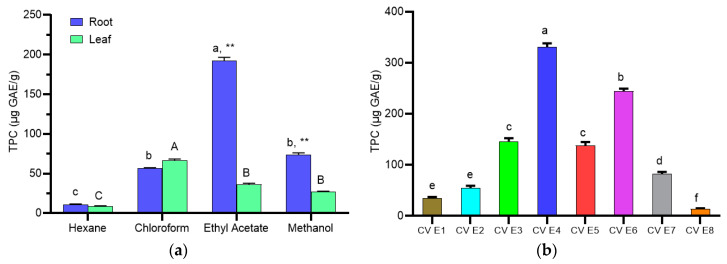
Total phenolic content in the (**a**) root and leaf crude extracts and (**b**) ethyl acetate root fractions of *C. vespertilionis*. All values are expressed as mean ± SD and are significantly different at *p* < 0.05 when compared between each group.

The Folin–Ciocalteu is a colorimetric assay that is based on the principle of electron transfer between the reagent and phenolic compounds present. Different intensities of blues produced depend on the reducing capacities are proportional to the total hydroxyl group of phenolic compounds thus, expressed as phenolic content [22]. The total phenolic content present in the extracts and fraction of *C. vespertilionis* are presented in Figure 3. The total phenolic content in the crude extracts of *C. vespertilionis* showed a varying ranged between 8.32 and 192.12 μg GAE/g. The highest amount of total phenolic content was found in the root extracts of ethyl acetate. In the ethyl acetate root fractions, the total phenolic content of F4 was found to be the highest at 330.92 μg GAE/g while the lowest phenolic content was shown in F8 at 13.56 μg GAE/g.

### 2.5. Antioxidant Properties of Crude Extracts and Fractions

#### 2.5.1. DPPH Free Radical Scavenging Activity

The DPPH assay is based on the ability of compounds from an extract or fraction to donate an electron. The purple stable organic nitrogen radical of DPPH is reduced to shades of yellow depending on the amount of hydrogen atoms received from the sample. The antioxidant strength of the *C. vespertilionis* extracts and fractions evaluated using the DPPH radical scavenging activity based on the IC_50_ values are tabulated in Table 2. The highest free-radical scavenging activity was seen in the ethyl acetate root extract and the F6 fraction. As natural standards, ascorbic acid (vitamin C) and α-tocopherol (vitamin E) were included to validate the accuracy of the assay by comparison with previous literature [23].

#### 2.5.2. β-Carotene Bleaching Assay

**Figure 4:**
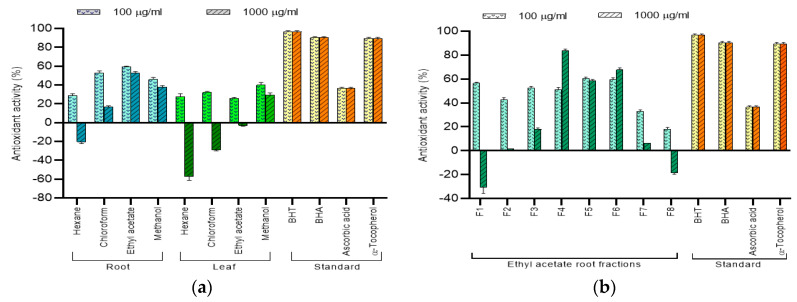
Antioxidant activity of *C. vespertilionis* (**a**) crude extract and (**b**) fractions evaluated by β-carotene lipid peroxidation assay at 100 and 1000 μg/mL.

The discoloration of β-carotene is affected by the presence of an antioxidant resulting in the formation of hydroperoxyls formed from linoleic acid as consistent heat is applied. As such, the evaluation of the extracts or fractions is based on the ability of the sample to exhibit antioxidative properties by neutralizing the peroxyl radicals and inhibiting β-carotene oxidation [23,24]. At both low and high concentrations, only the ethyl acetate root extract displayed good inhibition toward β-carotene oxidation (Figure 4a), accounting for 59.52% and 52.73%, respectively. The trend of the results suggests that the compounds responsible for the antioxidant activity are mainly found in the root of *C. vespertilionis*. Most fractions displayed better oxidative inhibition of β-carotene at low concentrations. At high concentrations, only F4 accounted for 83.68% inhibition of β-carotene bleaching which is almost comparable to the both the natural and synthetic standards. In addition, the results of the assay also revealed the hexane root extract; hexane, chloroform and ethyl acetate leaf extracts; F1 and F8 to show pro-oxidative tendencies at high concentrations which are in agreement with Zhang and Omaye [25].

### 2.6. Chemical Profiling of Active Fraction Using LC-MS/MS

**Figure 5:**
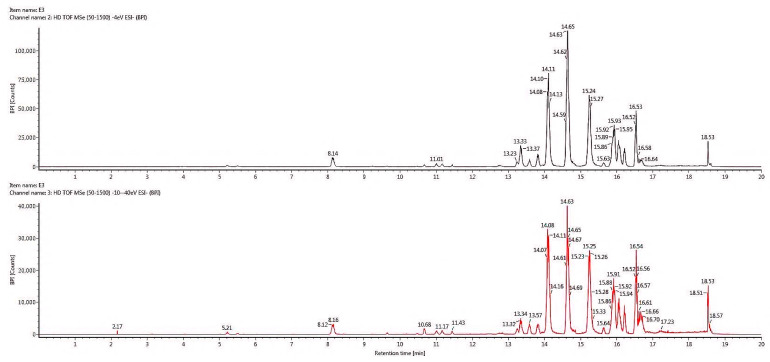
LC-chromatogram in negative ion mode for BPI plot of ethyl acetate root extract F3.

**Figure 6:**
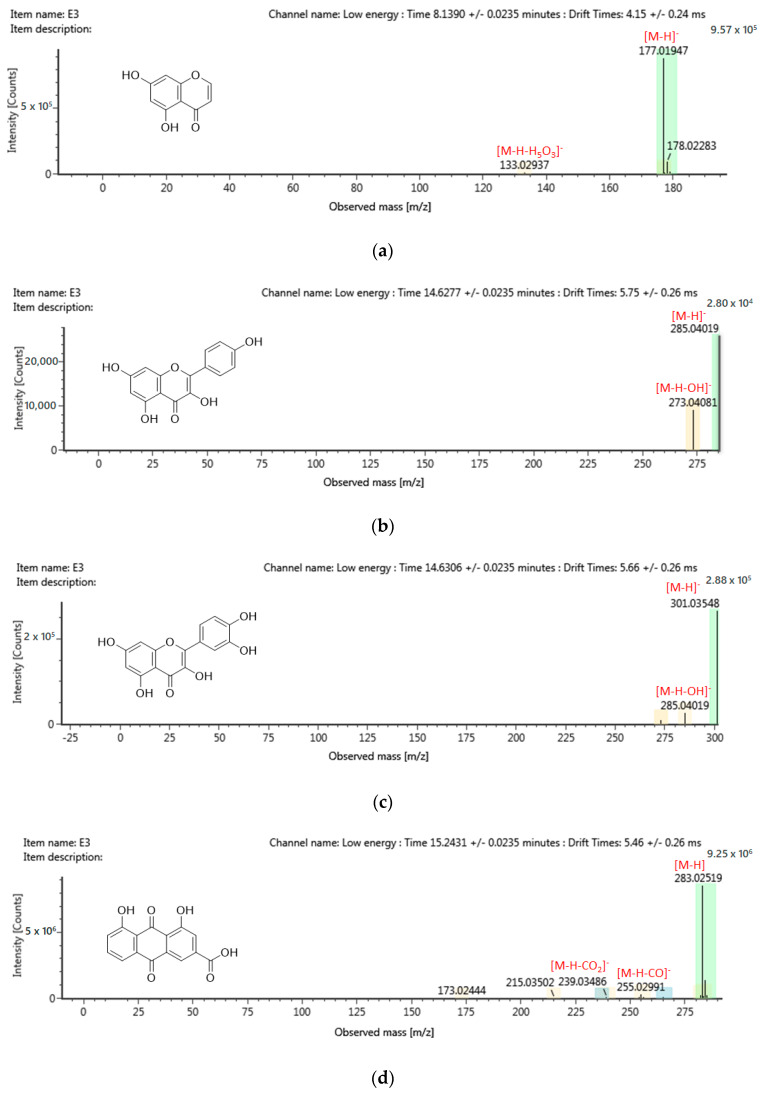

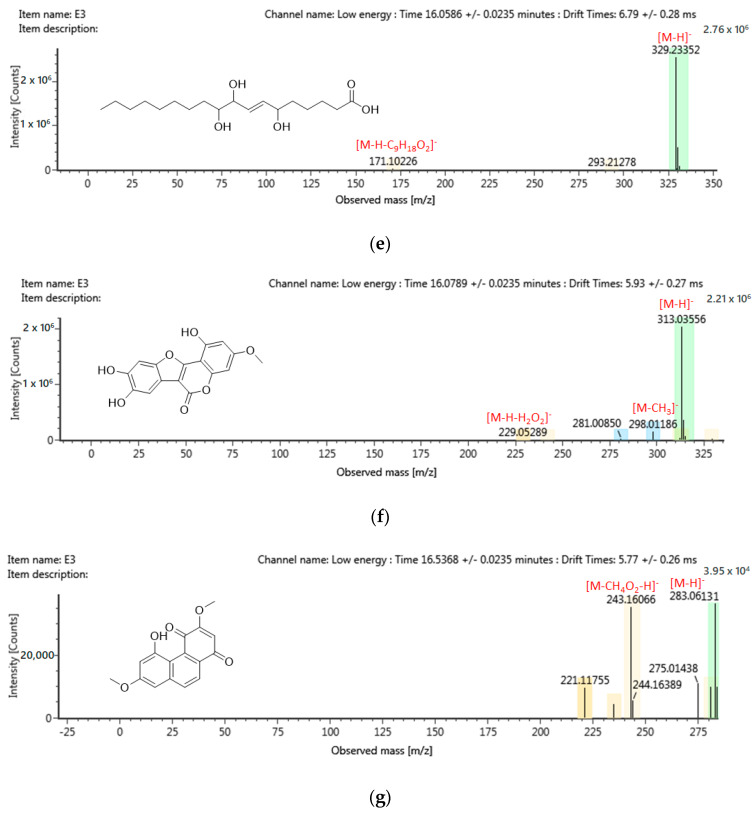
MS/MS spectra fragmentation of tentatively assigned compounds with potential anti-cancer properties from F3 as (**a**) 5,7-Dihydroxy-chromone, (**b**) Kaempferol, (**c**) Quercetin, (**d**) Rhein, (**e**) Sanleng acid, (**f**) Wedelolacetone and (**g**) Denbinobin.

The advancement of merging technologies introduces the current trend of phytocompound analysis using the LC-MS/MS fragmentation and ChemSpider database, thus, allowing the identification of unknown and known compounds in the plant extract. Figure 5 shows the most active fraction, F3, with a tentative identification of 205 compounds. The identity and grouping of the assigned compounds are based on the accuracy of the mass, chromatographic behavior, consecutive MS analyses, matching molecular mass of parent ions and m/z. From the tentative identification of 205 compounds, only those with high abundance are listed in Table 3 according to the group of secondary metabolites. A total of seven compounds that were reported to possess anti-cancer properties were further analyzed for their MS/MS spectra fragmentation to confirm the identity of the structure suggested (Figure 6).

## 3. Discussion

Breast cancer is amongst the most life-threatening cancers among women. Despite the continual advancement of medical technologies for cancer treatment, the increasing number of deaths has breast cancers almost categorized as a chronic disease. Most deaths are associated with metastases, some experience poor prognosis while others succumb to the side effects of current therapeutic strategies. Over the years, many researchers have ventured into natural products and their potential as their bioactive compounds are naturally-occurring and may possibly exhibit better efficacy and safety compared to synthetic chemotherapy drugs. In fact, many plants have been investigated and reported to exhibit anti-breast cancer properties. *C. vespertilionis* is a medicinal plant prized for its anti-inflammatory and anti-cancer properties. At present, the potential of *C. vespertilionis* as an alternative medicine specifically for breast cancers have yet to be reported. In addition, very little is also known regarding the active phytocompounds that are responsible toward the bioactivity of the plant. The study investigated the anti-breast cancer potential of *C. vespertilionis* root and leaf extracts and active fractions on two human breast cancer cell lines: MDA-MB-231 and MCF-7. The phytocompounds that were responsible toward the cytotoxic effects were narrowed down using the TPC assay and a correlation study between the phenolic content and its antioxidant activity. The most active fraction was then characterized using the LC-MS/MS.

From the cytotoxic studies conducted, it was suggested that the ethyl acetate root extract of *C. vespertilionis* contained phytocompounds that are responsible for the cytotoxicity against breast cancer cell lines. One study suggests the capability of polar solvents such as dichloromethane and ethyl acetate to extract phytocompounds such as polyphenols and terpenes which are known to exhibit good anticancer and antioxidant properties [11]. In addition, the results of this study are in agreement with Hofer [8] where the ethyl acetate fraction of *C. vespertilionis* showed significant anti-proliferative and pro-apoptotic effect against the medullary thyroid carcinoma and neuroendocrine cancer cell lines. According to the criteria established by the National Cancer Institute (NCI) Plant Screening Program, the extract of a medicinal plant is considered to have potential if the in-vitro cytotoxicity studies reported an IC_50_ value of less than 20 μg/mL following incubation between 48–72 h [26]. The ethyl acetate root extract demonstrated the lowest IC_50_ value compared to other extracts (*p* < 0.05) against MDA-MB-231 and MCF-7 cell lines. Furthermore, the extract also showed selective cytotoxicity toward normal cell lines [27].

Therefore, the ethyl acetate root extract was selected for further fractionation and the results suggested the potential of F3 as an anti-breast cancer agent. Although the cytotoxicity of the F3 against both the cancer cell lines showed no significant difference, the ethyl acetate crude extracts showed better inhibitory effects against MDA-MB-231 cells. The difference of bioactivity in the crude extract suggests the different apoptosis mechanism affected by the presence of phytocompounds in the extract. According to previous studies, cell metabolism of different phytocompounds varies between cell lines. Flavonoids were predominantly metabolized via the CYP1B1-mediated pathway in MDA-MB-231, while MCF-7 was metabolized via the CYP1A1-mediated pathway [28]. Another study observed the apoptosis of breast cancer cells against triterpenes and found the cell death mechanism in MDA-MB-231 to be P53 independent, while MCF-7 demonstrated P53 signaling dependence [29]. In addition, polyketides were also reported to activate the mitochondrial apoptotic pathway in MDA-MB-231 but induced the ERα-related apoptotic pathway in MCF-7 cells [30]. In the selectivity index study, it was interesting to note that compared to the selectivity of doxorubicin, the SI values of F3 recorded 4.4 and 3.3 times higher in the MDA-MB-231 and MCF-7 cell line. Although doxorubicin exhibited high cytotoxicity at very low concentrations, it appears to be harmful toward both the cancer and healthy cells which explains the low value of selectivity. In comparison, although the root extracts and fractions required a higher concentration to inhibit 50% of the cancer cells, a higher concentration, between 3–8 times, was required to inhibit 50% of the healthy cells. Therefore, F3 was suggested as a potential alternative treatment for breast cancer due to the selectivity shown against the different breast cancer cell lines.

Polyphenolics are mostly responsible for the pigmentation, growth and protection of a plant with the tendency to exhibit good anti-inflammatory, anti-cancer, antiviral and antioxidant properties. This is mainly due to their phytoalexin properties and strong astringency. The bioactivity of an extract is also dependent on the functional group bonded to the phenolic aromatic ring and the availability of free hydroxyl groups. This gives polyphenols strong reducing properties which promote good antioxidant capabilities [31,32,33]. According to Hyun et al. [34] the antioxidant properties of an extract is highly attributed to its high total phenolic content. In addition, compounds with high antioxidative strength are able to lower the production of reactive oxygen species (ROS), thus minimizing potential cellular damage and preventing cancer [35].

From the results, it was seen that the TPC assay, cytotoxicity and antioxidant results for the crude extract are in agreement. The results shown by the ethyl acetate root extract of *C. vespertilionis* are in agreement with Barchan and Arakrak [24], where the antioxidant strength demonstrated a positive correlation to the total phenolic content present in the extract. In addition, the results also suggest the presence of flavonoids, flavones, phenolic acids, terpenoids and tannins as these secondary metabolites are known to possess free hydroxyl groups [32]. Interestingly, the results of the fractions did not agree with each other. While F3 showed the best cytotoxicity, the highest TPC and β-carotene lipid peroxidation was however demonstrated by F4 and the highest DPPH was demonstrated by F6. This suggests that the cytotoxic effects of F3 could be due to other phytocompounds class besides polyphenols. The antioxidant patterns in fractions tend to vary with the process of isolation. Furthermore, the nature of mixed polarity solvents used in the isolation process also influences their phenolic components and a fraction’s antioxidant strength [36,37,38]. The results of the fractions, however, agree with Noreen [39] where fractions eluted from a more polar solvent mixture tend to exhibit better antioxidant properties, although other bioactivity results may differ.

Secondary plant metabolite has been known to exhibit a wide range of pharmacological properties including anti-cancer and anti-inflammatory. Flavonoids, saponins, alkaloids and terpenoids are common phytochemicals associated with the development of drug and supplements. The tentative assignment of compounds (Table 3) suggests the presence of flavonoids, coumarins and quinone. Polyphenols especially flavonoids and coumarins have been reported to demonstrate anti-proliferative effects as well as high antioxidative strength while quinone, a minor class compound is also valued for its high antioxidant and anti-cancer properties, especially toward breast cancers [28,40,41]. The finding suggests that the significant cytotoxic effects reported in both the active extract and fraction was due to presence of polyphenolic compounds. It was also suggested that the bioactivity of the phytocompounds present in the ethyl acetate root extract may be part of a synergistic combination as the cytotoxicity, TPC and antioxidant properties are no longer in agreement with each other in its fractions.

In conclusion, the present study revealed that the roots of the *C. vespertilionis* showed better bioactivity compared to the leaves of the plant. Specifically, the ethyl acetate root extract of *C. vespertilionis* was seen to possess strong cytotoxic effects against breast cancer cell lines especially against the triple negative breast cancer cell line, MDA-MB-231. In addition, the ethyl acetate root extract also exhibited significant antioxidant capabilities with high phenolic content. Although the cytotoxicity of the fractions did not agree with the antioxidant properties, further characterization suggests the presence of flavonoids, coumarins and quinones as phytocompounds found within the extract and fraction that may have contributed toward its anti-breast cancer and antioxidant properties. Even so, the study provided a new insight toward the potential of *C. vespertilionis* as an anti-breast cancer agent.

## 4. Materials and Methods

### 4.1. Plant Material

Roots and leaves specimen of the *Christia vespertilionis* were supplied by Seribu Satu Herba Enterprise, Malaysia. The herbarium voucher specimen (SK 2247/13) was identified, authenticated and kept at the Biodiversity Unit of the Institute of Bioscience, Universiti Putra Malaysia. The roots were sun dried and chopped into one inch lengths while the leaves were shade dried. Both parts were ground into fine powder.

### 4.2. Chemical Reagents

Chemicals were purchased from Fisher Scientific (Loughborough, Leicestershire, UK); RPMI-1640 with L-Gln (Roswell Park Memorial Institute Medium), penicillin-streptomycin mixed solution (stabilized) and 0.25%-trypsin solution were purchased from Nacalai Tesque Inc. (Kyoto, Japan); 2,2-diphenyl-2pocrylhydrazyl hydrate (DPPH), Folin-Ciocalteu’s phenol reagent, Tween 20, α-tocopherol, ascorbic acid, 3-(4,5-dimethylthiazol-2-yl)-2,5-diphenyltetrazolium bromide (MTT) powder and trypan blue dye solution were purchased from Sigma-Aldrich (St. Louis, MO, USA); gallic acid was obtained from Fluka Biochemica (Buchs, Switzerland); fetal bovine serum (FBS) (EU approved) was purchased from Tico Europe Ltd. (Amstelveen, The Netherlands, Europe); dimethyl sulfoxide analytical grade (DMSO) and phosphate buffered saline tablet (PBS) was purchased from Millipore Sigma (Burlington, MA, USA).

### 4.3. Preparation of Plant Extracts and Fractionation

**Figure 7:**
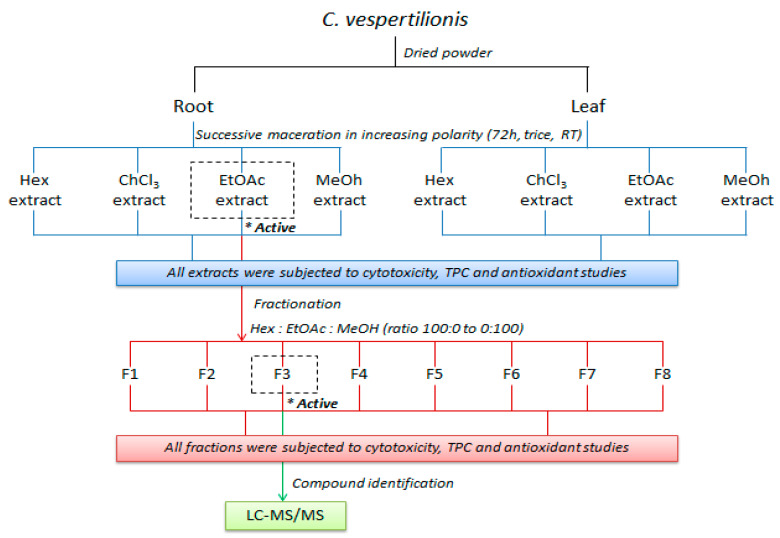
Schematic diagram of the extraction and fractionation of *C. vespertilionis*.

The fine powders of the plant material were subjected to successive solvent extraction by the conventional cold maceration method as described [42]. Briefly, 1 kg of the plant samples (root and leaf) was soaked in succession with 4L of hexane, chloroform, ethyl acetate, and methanol in increasing polarity thrice for 72 h. The mixture was filtered through Whatmann filter paper No. 1 and concentrated under reduced pressure to produce crude extracts. The remaining residue was re-extracted trice before moving to the next polar solvent. The extraction of the roots of *C. vespertilionis* afforded a yellowish green residue of hexane extract (3.5 g), a dark green residue of chloroform extract (5.5 g), a reddish brown residue of ethyl acetate extract (4.4 g) and a very dark brown residue of methanol extract (25.2 g). The extraction of the leaves gave a very dark green residue of hexane (10.4 g), chloroform (12.7 g), ethyl acetate (6.3 g) and methanol extract (41.9 g). The extracts were completely dried under a fume hood, stored at −20 °C and dissolved in DMSO for biological assays as needed. The most active extract was subjected to further fractionation by a column chromatography (6.0 × 20 cm) over silica gel (230–400 mesh ASTM, Merck Kieselgel 60 PF254 No. 9385, Merck & Co., Kenilworth, NJ, USA). The column was eluted with hexane-ethyl acetate-methanol solvent system in the order of increasing polarity to yield eight fractions (F1–F8). The graphical methodology shown in Figure 7 represents the flow of the experiment conducted in this study.

### 4.4. Cell Culture

The triple-negative human breast adenocarcinoma (MDA-MB-231), hormone receptor positive human breast adenocarcinoma (MCF-7) and normal mouse embryo fibroblast (NiH/3T3) cell lines were purchased from the American Type and Culture Collection (ATCC) (Rockville, MD, USA). All the cell lines were cultured in RPMI-1640 supplemented with 10% FBS and 1% antibiotics (100 IU/mL penicillin and 100 μg/mL streptomycin). The cells were maintained at 37 °C incubator in a humidified atmosphere of 5% CO_2_.

### 4.5. Determination of Cell Viability

The MTT colorimetric assay previously described was used to measure the cytotoxicity of the extracts against breast cancer cell lines [43]. Briefly, the cells were counted and seeded (1 × 10^5^ cells/mL) in a 96-well flat bottom microplate followed by 24-h incubation. Subsequently, the medium was removed and the cells were treated with different concentrations of *C. vespertilionis* extracts (3.13–200 μg/mL). The untreated group (control) was also included. Following the 72-h incubation, the treatment was removed and fresh growth medium was added. Then, 20 μL of MTT solution (5 mg/mL) was added into each well and further incubated for 3 h in a humidified incubator of 5% CO_2_ atmosphere at 37° in the dark. The supernatant was then aspirated and 100 μL of DMSO was added. The plates were slightly shaken to completely dissolve the dark-blue formazan crystals. The absorbance was measured at 570 nm using Bio-Tek μQuant microplate reader (Biotek Instrument, Winooski, VT, USA). The percentage of cell viability was calculated as shown in Equation (1) [44]:

(1) $$\mathrm{Cell}\;\mathrm{viability}=\frac{\left(OD_{treated}-OD_{blank}\right)}{\left(OD_{control}-OD_{blank}\right)}\times 10$$

The cytotoxicity of the extracts was expressed as IC_50_, the concentration of the extract that inhibits 50% of the cell viability compared to the control group.

### 4.6. Determination of Total Phenolic Content (TPC)

The total phenolic content of the root and leaf extract was determined using the Folin–Ciocalteu assay with slight modification [23]. The quantification of phenolic compounds is based on the external calibration using different concentrations of gallic acid (0–500 μg/mL). In brief, 40 μL of the extract or fraction (500 μg/mL) and the various concentrations of gallic acid solution were added into a 96-well plate. Then, 20 μL of folic reagent was added. After a 5-min incubation period at room temperature, 80 μL of Na_2_CO_3_ (60 mg/mL) followed by 60 μL of distilled water was added. The plates were incubated for 90 min in the dark and the absorbance was measured with a spectrophotometer at 760 nm. The TPC was calculated as μg gallic acid equivalent per gram of extract or fraction (μg GAE/g) with reference to the gallic acid calibration curve.

### 4.7. Determination of Antioxidant Activity

The DPPH and β-carotene-linoleate bleaching assay were used to evaluate the antioxidant activity of all the extracts and fractions. Stock solutions of the samples were prepared in methanol at 2 mg/mL concentration from dry weight. Further dilution with methanol was performed to provide different concentrations.

#### 4.7.1. DPPH Free Radical Scavenging Activity

The free radical scavenging assay was carried out using 2, 2-diphenyl-2-picrylhydrazyl (DPPH) radical with slight modifications [23]. Briefly, 30 μL of 0.1 mM DPPH^•^ solution was added to 70 μL of the tested extracts and fractions dissolved in methanol at various concentrations (31.25–2000 μg/mL) in a 96-well microplate. After 30 min of incubation in the dark at room temperature, the absorbance of the reaction was measured at 517 nm against a blank. The degree of free radical scavenging was measured using Equation (2) while the IC_50_ values were determined by the interpolation of non-linear regression analysis.

(2) $$\mathrm{DPPH}\;\mathrm{scavenging}\;\mathrm{activity}\;\left(\%\right)=\frac{A_{control}-A_{sample}}{A_{control}}\times 100$$

#### 4.7.2. β-Carotene Bleaching Assay

The antioxidant activity of *C. vespertilionis* root and leaf extract was evaluated by the β-carotene linoleate model system with slight modification [23]. Briefly, the working reagent containing 210 μL of β-carotene (1 mg/mL chloroform), 5 μL of linoleic acid and 42 μL of Tween-20 was prepared. The chloroform was evaporated under 40 °C using a rotary evaporator. Subsequently, distilled water (10 mL) was added into the mixture to form an emulsion. Then, 200 μL of emulsion was added into the 96-well microplates containing 50 μL of extract and fraction dissolved in methanol (1000 and 100 μg/mL). The absorbance reading at 470 nm was first taken at the start of the experiment (t = 0 h) and after an incubation in the dark at 50 °C for 2 h. The antioxidant activity (AA) was determined based on the rate of lipid peroxidation using Equation (3):

(3) $$\mathrm{AA}\;\left(\%\right)=\left\{1-\left[\frac{A_{t=0}-A_{t=2}}{A_{c=0}-A_{c=2}}\right]\;\right\}\times 100$$

where *A_t_*_=0_ and *A_c_*_=0_ are the absorbance values measured at 0 h of incubation for the sample and control, *A_t_*_=2_ and *A_c_*_=2_ are the absorbance value measured after 2 h of incubation for the sample and control, respectively.

### 4.8. LC-MS/MS Analysis

The analysis of the most active fraction of *C. vespertilionis* was performed using an ultra-performance liquid chromatography (UPLC) system (Waters, Milford, CT, USA). The conditions of the LC-MS/MS analysis are provided in Table 4.

### 4.9. Statistical Analysis

Data were analyzed using GraphPad Prism 8 (GraphPad Software, San Diego, CA, USA). All experiments were carried out in triplicate and all results were expressed as mean ± SD. Analyses of variances (ANOVA) were carried out to evaluate the significant difference followed by the Dunnet’s multiple comparison test. *p* < 0.05 was considered significant.

## Figures and Tables

**Table 1:** Selectivity index of the most active and second active extract and fraction of *C. vespertilionis* in comparison to drug control doxorubicin against breast cancer cell lines based on the IC_50_ values.

Cell Line	IC50 at 72 h Incubation (μg/mL)				
	Root Extract		Ethyl Acetate Fraction		Doxorubicin (DOX)
	Chloroform	Ethyl Acetate	F3	F4	
MDA-MB-231	29.58 ± 3.80	11.34 ±1.20	5.72 ± 0.99	12.53 ± 1.44	0.05 ± 0.01
MCF-7	54.55 ± 9.51	44.65 ± 5.78	8.98 ± 1.06	32.94 ± 2.72	0.06 ± 0.01
3T3	98.18 ± 13.35	77.38 ± 4.71	49.90 ± 8.63	68.30 ± 13.87	0.10 ± 0.01
SI 3T3/MDA	3.32 *	6.82 *	8.72 *	5.45 *	2.00
SI 3T3/MCF	1.80	1.73	5.56 *	2.07	1.67

All values are expressed as mean ± SD and values with asterisks are significant with selective index (SI) > 3.

**Table 2:** DPPH free radical scavenging activity of *C. vespertilionis* crude extracts, fractions and standards based on the IC_50_ values.

DPPH IC_50_ (μg/mL)			
	**Root Extract**		**Leaf Extract**
Hexane	>2000		>2000
Chloroform	338.07 ± 3.32 ^b^		679.43 ± 4.72 ^a,A^
Ethyl acetate	70.16 ± 1.49 ^a^		644.90 ± 20.09 ^a,A^
Methanol	421.73 ± 5.40 ^c^		716.37 ± 16.46 ^a,B^
**Ethyl Acetate Root Fraction**			
F1	>2000	F5	85.28 ± 1.13 ^a^
F2	1786 ± 14.73 ^e^	F6	76.71 ± 0.29 ^a^
F3	169.63 ± 4.89 ^b^	F7	235.63 ± 11.75 ^c^
F4	101.53 ± 1.47 ^a^	F8	1157.33 ± 22.50 ^d^
**Standards**			
Ascorbic acid	16.99 ± 1.22	α-tocopherol	10.49 ± 0.98

All values are expressed as mean ± SD and data with superscripts are significantly different at *p* < 0.05 when compared within each group. For the leaf extract, ^a^ represents no significance when comparing chloroform with ethyl acetate and methanol extract while ^A,B^ represents no significance when comparing ethyl acetate with chloroform but with significance when compared with methanol.

**Table 3:** Tentative assigned phytocompounds in the F3 fraction of *C. vespertilionis*.

RT (m/z)	Component Name	Formula	Observed Neutral Mass (mDa)	Mass Error (mDa)	Observed *m*/*z* (ppm)	Mass Error (ppm)	Response	Adducts	Total Fragment Found
**Flavonoids**									
13.34	3,4-Dihydro-4-(4′-hydroxyphenyl)-5,7-dihydroxycoumarin	C_15_H_12_O_5_	272.0685	0.1	271.0613	0.3	207136	[M − H]^−^	9
13.81	Sternbin	C_16_H_14_O_6_	302.0792	0.2	301.0719	0.6	118792	[M − H]^−^	13
14.63	Kaempferol	C_15_H_10_O_6_	286.0475	−0.3	285.0402	−1.0	2817	[M − H]^−^	6
14.63	Quercetin	C_15_H_10_O_7_	301.0428	0.1	301.00828	0.4	29738	[M − H]^−^	3
14.64	Kuchecarpins C	C_17_H_16_O_7_	332.0901	0.5	331.0828	1.5	1483888	[M − H]^−^	62
16.54	8-C-Prenyl kaempferol	C_20_H_18_O_6_	354.1107	0.1	353.1034	1.0	104746	[M − H]^−^	46
16.54	Kuwanon L	C_35_H_30_O_11_	626.1796	0.8	625.1723	1.3	22479	[M − H]^−^	67
**Quinones**									
14.10	Fallacinol	C_16_H_12_O_6_	300.0637	0.3	299.0564	0.9	1026234	[M − H]^−^	9
15.24	Alizarin	C_14_H_8_O_4_	240.0421	−0.1	239.0349	−0.5	5774	[M − H]^−^	1
15.24	Purpurin	C_14_H_8_O_5_	256.0372	1.0	255.0299	0.1	27010	[M − H]^−^	2
15.24	Rhein	C_15_H_8_O_6_	284.0325	0.4	283.0252	1.3	925958	[M − H]^−^	8
16.54	Denbinobin	C_16_H_12_O_5_	284.0686	0.1	283.0613	0.4	3957	[M − H]^−^	10
**Coumarins**									
8.14	5,7-Dihydroxychromone	C_9_H_6_O_4_	178.0267	0.1	177.0195	0.8	75787	[M − H]^−^	4
16.08	Wedelolacetone	C_15_H_12_O_5_	314.0428	0.2	313.0356	0.6	232941	[M − H]^−^	8
**Phenolic acids**									
15.92	Sanleng acid	C_18_H_34_O_5_	330.2408	0.2	329.2335	0.5	683016	[M − H]^−^	17

**Table 4:** LC-MS/MS analysis condition.

Parameter		Condition		
**Ultra-Performance Liquid Chromatography (UPLC)**		**ACQUITY UPLC HSS T3**		
**Column**		100 mm × 2.1 mm × 1.8 μm, Waters		
**Column Temperature**		40 °C		
**Flow Rate**		0.6 mL/min		
		A (water + 0.1% formic acid)		
		B (acetonitrile + 0.1% formic acid)		
**Mobile Phase**		**Time**	**A (%)**	**B (%)**
		0	99	1
		5	99	1
		16	65	35
		18	0	100
		20	99	1
**Injection Volume**		1 μL		
**Metabolite Eluted**		Vion IMS HDMS QTOF (Waters), positive and negative		
Ion source	capillary voltage (kV)	1.5		
	reference capillary voltage (kV)	3		
Collision energies	low-energy (eV)	4		
	high-energy (eV)	10–40		
Scan range (Da)		50–1500		
Scan time (s)		0.1		
